# Targeting the Parasite's DNA with Methyltriazenyl Purine Analogs Is a Safe, Selective, and Efficacious Antitrypanosomal Strategy

**DOI:** 10.1128/AAC.00596-15

**Published:** 2015-10-13

**Authors:** Boris Rodenko, Martin J. Wanner, Abdulsalam A. M. Alkhaldi, Godwin U. Ebiloma, Rebecca L. Barnes, Marcel Kaiser, Reto Brun, Richard McCulloch, Gerrit-Jan Koomen, Harry P. de Koning

**Affiliations:** a College of Medical, Veterinary and Life Sciences, Institute of Infection, Immunity and Inflammation, University of Glasgow, Glasgow, United Kingdom; b Wellcome Trust Centre for Molecular Parasitology, University of Glasgow, Glasgow, United Kingdom; c Van 't Hoff Institute for Molecular Sciences, University of Amsterdam, Amsterdam, The Netherlands; d Department of Biology, College of Science, Aljouf University, Skaka, Kingdom of Saudi Arabia; e Swiss Tropical and Public Health Institute, Basel, Switzerland; f University of Basel, Basel, Switzerland

## Abstract

The human and veterinary disease complex known as African trypanosomiasis continues to inflict significant global morbidity, mortality, and economic hardship. Drug resistance and toxic side effects of old drugs call for novel and unorthodox strategies for new and safe treatment options. We designed methyltriazenyl purine prodrugs to be rapidly and selectively internalized by the parasite, after which they disintegrate into a nontoxic and naturally occurring purine nucleobase, a simple triazene-stabilizing group, and the active toxin: a methyldiazonium cation capable of damaging DNA by alkylation. We identified 2-(3-acetyl-3-methyltriazen-1-yl)-6-hydroxypurine (compound 1) as a new lead compound, which showed submicromolar potency against Trypanosoma brucei, with a selectivity index of >500, and it demonstrated a curative effect in animal models of acute trypanosomiasis. We investigated the mechanism of action of this lead compound and showed that this molecule has significantly higher affinity for parasites over mammalian nucleobase transporters, and it does not show cross-resistance with current first-line drugs. Once selectively accumulated inside the parasite, the prodrug releases a DNA-damaging methyldiazonium cation. We propose that ensuing futile cycles of attempted mismatch repair then lead to G_2_/M phase arrest and eventually cell death, as evidenced by the reduced efficacy of this purine analog against a mismatch repair-deficient (*MSH2*^−/−^) trypanosome cell line. The observed absence of genotoxicity, hepatotoxicity, and cytotoxicity against mammalian cells revitalizes the idea of pursuing parasite-selective DNA alkylators as a safe chemotherapeutic option for the treatment of human and animal trypanosomiasis.

## INTRODUCTION

African trypanosomiasis covers a complex of diseases in humans (sleeping sickness), cattle (nagana), camels (surra), horses (dourine), other livestock, and in wild and companion animals (1). Transmission of the human disease is entirely limited to the habitat of the tsetse fly vector in sub-Saharan Africa and is caused by two subspecies of Trypanosoma brucei: T. brucei gambiense in West and Central Africa, and T. brucei rhodesiense in East and southern Africa. T. brucei rhodesiense is a well-known zoonosis, and several other species, including T. brucei brucei, Trypanosoma congolense, Trypanosoma equiperdum, Trypanosoma evansi, and Trypanosoma vivax, give rise to a range of animal pathologies. As T. equiperdum, T. evansii, and T. vivax do not require tsetse fly transmission, these infections have spread to other regions, including the Middle East, southern Asia, and South America (2, 3).

Virtually the only method of control for human African trypanosomiasis is treatment with chemotherapy, but the few drugs available are old, often toxic, and threatened by resistance (4). Due to the introduction of nifurtimox-eflornithine combination therapy (NECT) for late-stage T. brucei gambiense human African trypanosomiasis (HAT), melarsoprol usage is finally being phased out in much of central Africa, but NECT is expensive and relies on a large number of intravenous infusions (5). For animal trypanosomiasis, the situation is almost as bad, with a few 50-year-old drugs for which resistance has been reported throughout Africa (4, 6). As there is no realistic prospect of a vaccine against any of the African Trypanosoma species, it follows that new drugs for African trypanosomiasis are urgently required. A few compounds are proceeding toward (pre)clinical test stages (7), but the success of these compounds is by no means ensured. In addition, since trypanosomiasis is a highly complex set of conditions of numerous infective species, hosts, and disease stages, it is highly unlikely that a single new compound can meet all the diverse needs. However, it is possible to demand that new trypanocides, in order to be of value, have lower toxicity than that of the current drugs, and, crucially, must be active against strains resistant to the current therapies, particularly diamidines and melaminophenyl arsenicals. The mechanisms of resistance and cross-resistance to and between these drugs have intensively been studied in the last decade, particularly in the model organism T. brucei brucei (8, 9). The activity and resistance of these trypanocides depend on a set of transport proteins that mediate their uptake into the trypanosomes: the aminopurine transporter P2/TbAT1 (10), the high-affinity pentamidine transporter 1 (HAPT1), encoded by aquaglyceroporin 2 (11, 12), and the low-affinity pentamidine transporter (LAPT1) (13).

The various diamidines and arsenicals are transported to different extents and with very different affinities by each of the three known drug transporters, explaining the complicated cross-resistance patterns (8).

Thus, dependence on one or more of the known drug transporters would almost certainly result in cross-resistance with existing chemotherapeutic agents. Yet, selective trypanocidal action is most commonly achieved through selective uptake, exploiting the extensive, unique, and highly efficient collection of transporter proteins of the trypanosome (14, 15). We therefore sought to rationally exploit a different set of high-affinity trypanosomal transporters, thereby ensuring rapid and selective accumulation of the agent within its target cell without building in cross-resistance to existing chemotherapy. The purine nucleobase transporters are perfectly suited for drug delivery, in that multiple variants of these transporters are expressed in trypanosomes, with overlapping substrate selectivities and much higher substrate affinities than those of their mammalian orthologues (16). In addition, T. brucei nucleobase transporters are proton symporters, meaning that they utilize the proton motive force across the plasma membrane to actively transport their substrate into the cell, even against a strong concentration gradient (17, 18). All this has the effect of rapidly and selectively pumping the active compound into the target cell and, as active transport is monodirectional, the substrate will not be able to egress from the cell in the same way (14).

The active toxophore to be carried through the nucleobase transporters, coupled to a nucleobase, must be in the right position of the purine ring and must be small enough not to interfere with translocation by the transporters. Here, we present the development of purine derivatives that meet these requirements by carrying a methyltriazenyl toxophore on the purine 2 position. These methyltriazenyl purines (MTPs) constitute a class of highly effective antitrypanosomal agents. We propose that this therapeutic class of agents damages the DNA of the parasite, which results in futile cycling of the mismatch repair system, leading eventually to parasite cell death.

## MATERIALS AND METHODS

### Cells.

Bloodstream-form T. brucei brucei strain 427 (BS221) (19), the derived multidrug-resistant clonal lines *tbat1*^−/−^ (10) and *MSH2*^+/−^, *MSH2*^−/−^ and *MSH2*^−/−/+^ (20, 21), and human embryonic kidney cells (HEK 293T) (22) were maintained and used for alamarBlue drug sensitivity assays (23), as described previously (24). For HEK 293T cells, an extended alamarBlue protocol was used, which was adapted from that described previously (22). Briefly, in a 96-well microtiter plate, cells were seeded at 30,000 cells in 100 μl of Dulbecco's modified Eagle's medium (DMEM) per well and allowed to adhere for 24 h. Serial drug dilutions were then added (100 μl per well), and after 30 h of incubation, alamarBlue reagent (20 μl) was added, and the plate was read after another 24 h of incubation. Assays for transport of [^3^H]hypoxanthine (at 0.05 μM for 60 s, 28 Ci/mmol; Amersham) by bloodstream-form T. brucei brucei strain 427 and for the transport of [2,8-^3^H]adenine (at 1 μM for 5 s, 24 Ci/mmol; PerkinElmer) by human red blood cells, obtained from whole-blood samples donated by healthy volunteers, was performed exactly as described previously using a rapid oil-stop protocol (19). For scanning electron microscopy, 2 × 10^6^ drug-treated bloodstream-form T. brucei brucei cells were washed twice with wash buffer (33 mM HEPES, 98 mM NaCl, 4.6 mM KCl, 0.55 mM CaCl_2_, 0.07 mM MgSO_4_, 5.8 mM NaH_2_PO_4_, 0.3 mM MgCl_2_, 23 mM NaHCO_3_, 14 mM glucose [pH 7.3]) and fixed with 2.5% glutaraldehyde in 0.1 M phosphate buffer (pH 7.4) for 1 h. The fixed cells were washed four times with 0.1 M sodium phosphate (pH 7.4) and placed on coverslips, which were immersed in osmium tetroxide (1% [wt/vol]) in distilled water for 1 h and then rinsed twice in distilled water. Samples were dehydrated through graded ethanol solutions, dipped in hexamethyldisilazane (HMDS) for 30 s, and left in a desiccator overnight. Dried samples were gold conductive coated using a sputter coater and viewed in a Philips 500 scanning electron microscope (EM) at 6 kV.

### DNA content and integrity.

The DNA content of bloodstream-form T. brucei brucei 427 treated with drugs was visualized by DAPI (4′,6-diamidino-2-phenylindole) staining followed by microscopic analysis, and by propidium iodide staining followed by flow cytometry, as described previously (25). The occurrence of DNA strand breaks in bloodstream-form T. brucei brucei 427 or HEK293T cells as a consequence of drug treatment was monitored by flow cytometry using the APO-BrdU TUNEL assay kit (catalog no. A23210; Life Technologies), according to the manufacturer's protocol.

### *In vivo* trypanocidal assays.

The experiments were performed essentially as described previously (26). Female NMRI mice were infected intraperitoneally (i.p.) with 10^4^ bloodstream-form T. brucei brucei (strain STIB 795) or 3 × 10^3^ T. brucei rhodesiense (strain STIB 900). Experimental groups of four mice were treated i.p. with test drug daily on four consecutive days starting from day 3 postinfection. A control group was infected but remained untreated. The tail blood of each mouse was checked for parasitemia up to 60 days postinfection; mice were culled when parasitemia was >10^8^ trypanosomes/ml of blood. Surviving and aparasitemic mice at day 60 were considered cured and were euthanized. All protocols and procedures used in this study were reviewed and approved by the local veterinary authorities of the Canton Basel-Stadt, Switzerland.

## RESULTS

### Rational design of methyltriazenyl purines for selective uptake by T. brucei.

**FIG 1:**
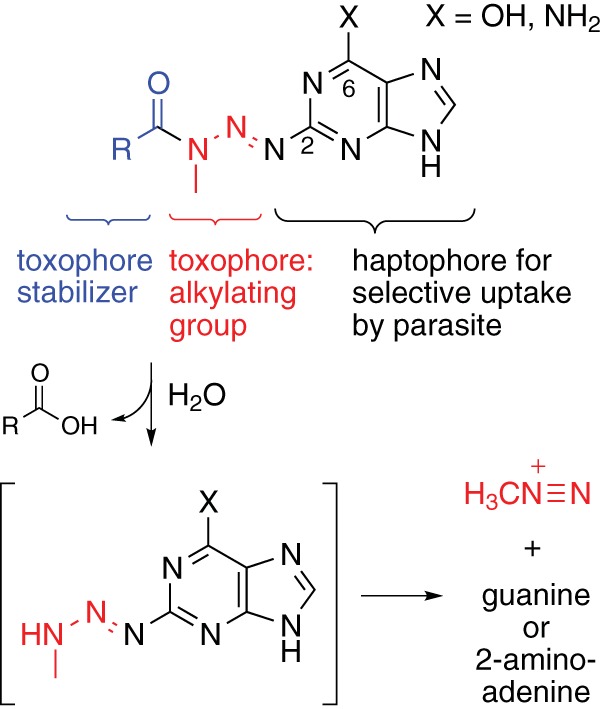
Design of methyltriazenyl purine antitrypanosomal prodrugs releasing methyldiazonium cations. Purine numbering is indicated.

In bloodstream form (BF), T. brucei purines are primarily taken up via the broad-specificity hypoxanthine transporters H2 and H3 (18). Substrate recognition models that we previously published for this transporter class showed that the purine 2-position is not utilized in binding to trypanosomatid nucleobase transporters, whereas it is engaged in hydrogen bond formation in human nucleobase transporters (19, 27). Substituents on the purine 6-position larger than the natural exocyclic heteroatoms were shown to significantly reduce affinity for the hypoxanthine H2 transporter (19). We therefore considered the purine 2-position to be most suitable for attaching a toxophore to the purine skeleton for its selective delivery into the parasite. To keep the size of the toxophore to a minimum, we opted for a simple methyltriazene moiety. The methyltriazenyl toxophore is spontaneously or enzymatically hydrolyzed in the body, causing the release of reactive methyldiazonium ions (28), which are known to cause fatal methylation of nucleobase moieties within nucleic acids. Examples of methyltriazenyl prodrugs (MTPs) are temozolomide and dacarbazine, which are used for the treatment of malignancies, including glioblastoma and metastatic melanoma (29, 30). We generated MTP derivatives designed to disintegrate by hydrolysis into three parts: (i) a purine nucleobase, specifically, guanine or 2-aminoadenine; (ii) a simple triazene-stabilizing group, such as a small carboxylic acid or an alcohol and CO_2_; and (iii) a methyldiazonium ion (Fig. 1, see supplemental material for synthetic details).

### Methyltriazenyl purines have potent antitrypanosomal activity.

**FIG 2:**
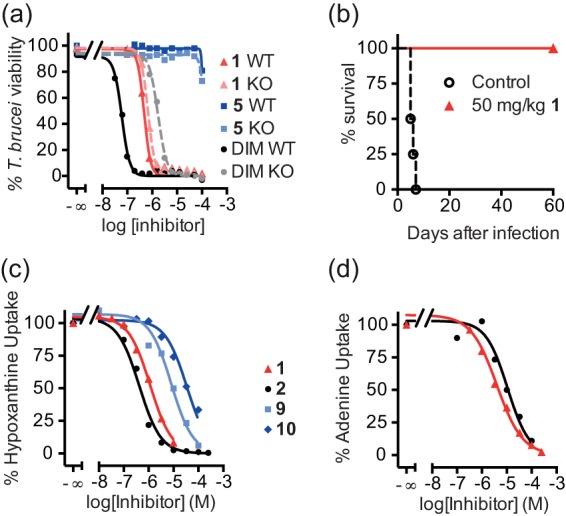
Methyltriazenyl purines demonstrate potent antitrypanosomal activity. (a) Dose-response curves for compound 1, compound 5 (a control lacking alkylating activity), and reference drug diminazene (DIM). Wild-type 427 (WT) BF T. brucei cells are compared with drug-resistant *tbat1*^−/−^ cells lacking the P2 purine transporter (knockout [KO]). The results from one representative experiment are shown from a minimum of 3 independent determinations (b) MTP compound 1 cures acute trypanosomiasis *in vivo*. Kaplan-Meier survival plot for female NMRI mice (*n* = 4 per group) after infection with T. brucei brucei (STIB 795) (inoculum, 1 × 10^4^ parasites). Intraperitoneal injection with compound 1 started 3 days after infection at a single dose of 50 mg/kg per day for 4 days. (c) Inhibition of the uptake of 0.05 μM [^3^H]hypoxanthine in BF T. brucei cells by the indicated purine analogs. (d) Inhibition of 1 μM [^3^H]adenine uptake in red blood cells by compounds 1 (black squares) and 2 (red triangles). (c and d) Data are the average and standard error of the mean (SEM) of triplicate determinations from one experiment performed in triplicate; each experiment was performed fully independently ≥3 times with highly similar outcomes. Average values for inhibition constants (*K_i_*) are given in Table 1.

An assessment of the antitrypanosomal activity of a series of MTPs (Table 1) identified MTP compound 1 with promising submicromolar potency against BF T. brucei *in vitro* (Fig. 2A). We found that the antitrypanosomal activity appeared to be affected by two main determinants: (i) the nature of the toxophore-stabilizing group that controls the rate of triazene hydrolysis, and (ii) the nature of the functional group on the purine 6-position. In general, 6-oxo MTPs appeared to have higher trypanocidal activity than that of 6-amino MTPs, with the exception of compound 4. Among the 6-oxo MTPs, the most active compounds were those with stabilizing electron-rich groups, such as acetamide (compound 1), phenylurethane (compound 2), and ethylurethane (compound 3), whereas compound 4, harboring a strongly electron-withdrawing *p*-nitrophenylurethane moiety, had practically lost antitrypanosomal activity. The two most potent 6-oxo MTPs, compound 1 and its derivative, compound 2, have hydrolysis half-lives of 6.8 ± 0.4 h and 5.6 ± 0.3 h, respectively, under physiological conditions (see Fig. S1 in the supplemental material), whereas *p*-nitrophenylurethanes, with hydrolysis half-lives in the order of 20 min (31), are probably too unstable and likely decompose before effective levels have been built up in the parasite. Among the 6-amino MTPs, the same trend was observed, with the derivative compound 6, containing an electron-rich toxophore-stabilizing group, showing higher trypanocidal activity than those with electron-withdrawing groups, such as compounds 7 and 8.

**TABLE 1:**
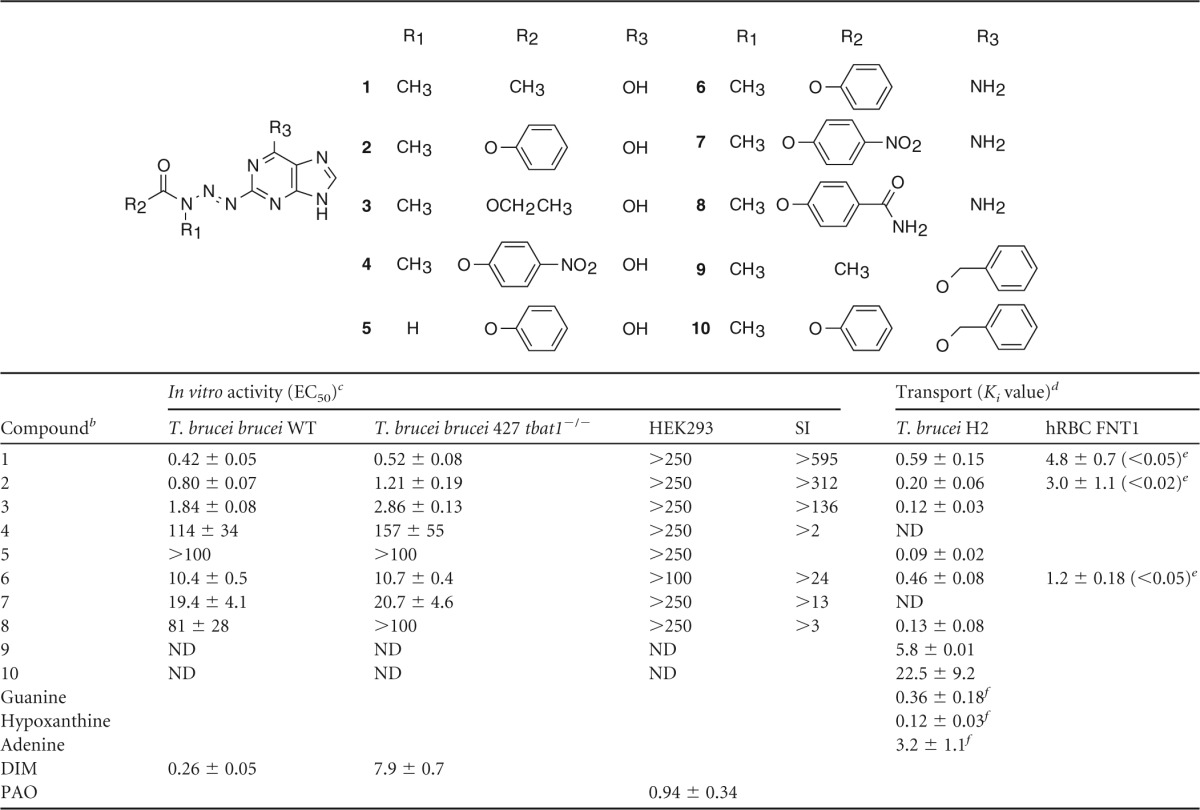
*In vitro* activity and transport parameters of trypanocidal methyltriazenyl purines^*a*^

a Data are in μM and represent the mean ± standard error of mean (*n* ≥ 3).

b DIM, diminazene, an antitrypanosomal reference drug. PAO, phenylarsine oxide, a general cytotoxic control.

c T. brucei brucei 427 *tbat1*^−/−^ lacks the TbAT1 transporter and displays no P2 transport activity (10). WT, wild type; HEK293, human embryonic kidney 293T cells; SI, *in vitro* selectivity index, calculated as EC_50_(HEK)/EC_50_(T. brucei brucei WT); ND, not determined.

d T. brucei H2, hypoxanthine transporter 2 of T. brucei brucei; hRBC FNT1, facilitative nucleobase transporter 1 of human red blood cells.

e Paired *t* test, measuring the significance of difference between T. brucei brucei H2 and hRBC FNT1 transport.

f Numbers taken from reference 18.

The higher trypanocidal activity of the 6-oxo derivative 2 versus the 6-amino derivative compound 6 may be partly due to higher translocation rates as a result of the higher affinity that 6-oxopurines typically have for trypanosomal nucleobase transporters (18, 19), resulting in an approximately 5-fold higher efficiency of transport for hypoxanthine over adenine (32). However, the 6-amino MTPs 6 (*K_i_* = 0.46 μM) and 8 (*K_i_* = 0.13 μM) rather surprisingly displayed a higher affinity for the H2 transporter than adenine (*K_i_* = 3.2 μM), whereas the 2-position triazene substitution had made no significant change to the affinity of the 6-oxo derivatives (Table 1). This observation suggests a slightly different binding orientation for the 6-amino and 6-oxo derivatives, as previously reported for substrates of the UapA nucleobase transporter of Aspergillus nidulans (33, 34). To assess potential cross-resistance with the current first-line trypanocidal drugs, we tested the MTPs against the drug-resistant clonal line T. brucei brucei 427 *tbat1*^−/−^, lacking the TbAT1/P2 aminopurine transporter (10) and thus displaying reduced sensitivity to diamidines and melaminophenyl arsenicals (4). The antitrypanosomal activities of the MTPs were not significantly different between the wild-type and *tbat1*^−/−^ cell lines, although the diamidine diminazene displayed 30-fold lower activity against *tbat1*^−/−^ cells (Fig. 2A and Table 1), indicating that cross-resistance with diamidines is not likely to occur (10, 35). Furthermore, these purine derivatives were not toxic to human embryonic kidney cells (HEK293T cells) to the maximum concentration tested (250 μM; Table 1). MTPs 1 and 2 displayed promising selectivity indices of >595 and >312, respectively. Control derivative compound 5, lacking methylating potential, did not show any toxic effects toward either trypanosomes or HEK cells, as expected. DNA-damaging agents are potentially mutagenic. However, an Ames test with compound 1 did not reveal any mutagenic activity before or after metabolic activation with induced rat liver homogenate S9 (see Fig. S2 in the supplemental material). Furthermore, compound 1 displayed no hepatotoxic effects on mammalian primary hepatocytes (see Fig. S3 in the supplemental material).

### Methyltriazenyl purine 1 cures acute trypanosomiasis *in vivo*.

Lead compound 1 showed a 100% cure rate in a mouse model of acute trypanosomiasis infection, in which each mouse was treated at 3 days postinfection by an intraperitoneal injection with 50 mg/kg of body weight of compound 1 for four consecutive days (Fig. 2B). All treated mice were parasite free after treatment, survived >60 days, and were considered cured. In the acute model, mice were infected with T. brucei brucei (STIB 795), which is pathogenic for rodents but not for humans. In a more stringent mouse model of trypanosomiasis, mice were infected with T. brucei rhodesiense (STIB 900), which causes HAT in eastern Africa. In this stringent model, treatment with 50 mg/kg compound 1 (i.p.) for four consecutive days showed a 75% cure rate (see Fig. S4 in the supplemental material).

### Selective uptake of MTPs by trypanosomes.

Having shown that MTPs are not likely to be taken up by TbAT1/P2 transport activity, we tested the affinity of MTPs for the H2 hypoxanthine transporter in BF T. brucei. MTPs 1 to 8 displayed high affinity for the H2 transporter, comparable to the affinities of the natural substrates hypoxanthine and guanine (Table 1). In line with our substrate recognition models (19), MTPs, such as compounds 1 and 2, lacking a 6-oxo substituent, showed higher affinity for the H2 transporter than their synthetic precursors, compounds 9 and 10, respectively, which still carry an *O*^6^-benzyl substitution (Fig. 2C). This likely also explains the much lower antiprotozoal potency exerted by the *O*^*6*^-benzyl-2-methyltriazenyl purines with antitumor activity (see Fig. S5 in the supplemental material) (31), consistent with the antiprotozoal activity being principally determined by effective uptake via the nucleobase transporters. Furthermore, antitrypanosomal MTPs 1 and 2 (Fig. 2D) displayed significantly higher affinity for the trypanosomal hypoxanthine H2 transporter than that for the human facilitative nucleobase transporter (FNT1) (Fig. 2D and Table 1), pointing to selective uptake by the parasite.

The standard culture medium, HMI-9, used for T. brucei drug sensitivity assays (but not for the hypoxanthine transport assays) contains 1 mM hypoxanthine, which might interfere with uptake of MTPs through hypoxanthine transporters during the 72 h of incubation. We therefore retested the potency of compound 1 on T. brucei grown in Creek's minimal medium (36) in the presence of either inosine (not a substrate of H2/H3 [18]) or hypoxanthine as the purine source (see Fig. S6 in the supplemental material). We found that the sensitivity of bloodstream-form T. brucei to compound 1 was indeed higher in a medium with inosine as the only purine source, compared to the same medium containing the same concentration of hypoxanthine (*P* < 0.02). The 50% effective concentrations (EC_50_s) for several reference drugs (pentamidine, suramin, and phenylarsine oxide) were not significantly different for the two purine sources. These observations are consistent with most of the uptake of compound 1 being mediated by hypoxanthine-sensitive transporters.

### Methyltriazenyl purine 1 causes cell cycle arrest in trypanosomes.

**FIG 3:**
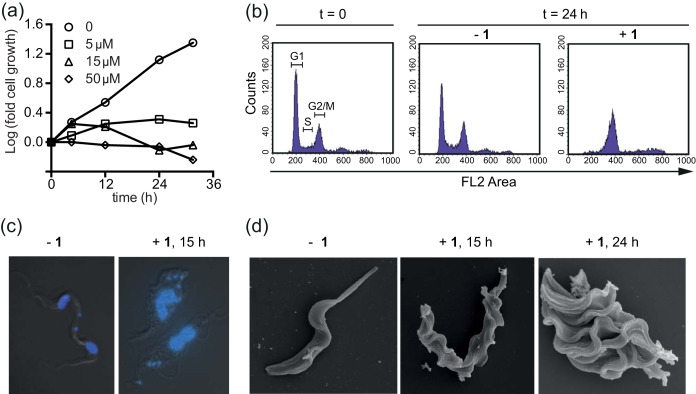
Methyltriazenyl purines cause cell cycle arrest in the G_2_/M phase in T. brucei. (a) Growth curves of BF T. brucei treated with the indicated doses of compound 1. (b) DNA content of BF T. brucei in the presence or absence of 15 μM compound 1, as determined by propidium iodide staining of fixed cells and measured by fluorescence-activated cell sorting (FACS). (c) DAPI staining of nuclear and kinetoplastid DNA of BF T. brucei cells exposed to 5 μM compound 1 (+1) or not (−1) for 15 h. (d) Scanning electron microscopy of wild-type trypanosomes incubated in normal medium (left) or medium containing 15 μM compound 1 for 15 h (middle) or 24 h (right).

Given its promising antiparasitic efficacy *in vitro* and *in vivo*, we selected lead compound 1 for further antitrypanosomal mechanism-of-action studies. A culture of BF T. brucei exposed to compound 1 was arrested in its proliferation (Fig. 3A). We analyzed the DNA content of these cells over 24 h by flow cytometry (Fig. 3B; see also Fig. S7 in the supplemental material). After 4 h of exposure to 15 μM compound 1, we observed a decrease in the proportion of cells with normal diploid (2C) DNA content, whereas an aberrant population of cells started to appear with a DNA content between 2C and 4C. After 24 h, this trend culminated in the majority of cells having an apparent 4C DNA content, indicating an inability to finish cell division after apparently completing DNA synthesis. We also monitored the karyotype distribution of BF T. brucei over time (see Fig. S8 in the supplemental material), using DAPI staining to visualize nuclei (N) and kinetoplasts (K). Within the first 8 h (the normal time to complete one cell cycle) of exposure to compound 1, we observed a significant increase in 1N2K cells, with a coincident decrease in 1N1K and 2N2K. After this time, the proportion of cells with 1N2K dropped, with a coincident increase in the proportion of cells having multiple kinetoplasts (1N3K, 1N4K increasing up to 1N8K). In the multikinetoplast cells, the nucleus was usually considerably enlarged and often bilobed, and cells typically showed aberrant morphology (Fig. 3C). Scanning electron microscopic (EM) analysis of cells treated with compound 1 showed that the aberrant cells grew multiple new flagella, accompanied by the ingression of various cleavage furrows (Fig. 3D). These results suggest that the cells are unable to complete nuclear division but do proceed with kinetoplast division independently, and cytokinesis does not occur. The absence of cytokinesis does not prevent kinetoplast replication and segregation or outgrowth of a new flagellum, which indicates that compound 1 predominantly affects replication and segregation of nuclear DNA.

### Mismatch repair is implicated in the mechanism of action.

**FIG 4:**
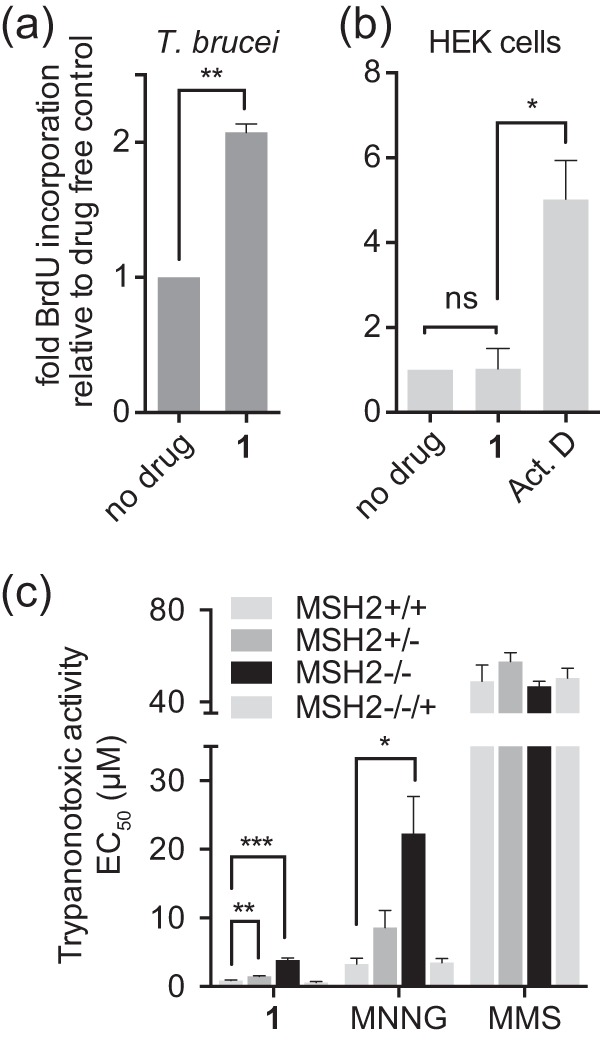
Trypanocidal activity of methyltriazenyl purines is linked to DNA damage and is mismatch repair dependent. (a) BF T. brucei brucei was exposed to compound 1 (3 μM) or left untreated. After 24 h, DNA damage was assessed by a TUNEL assay. BrdU incorporation was analyzed by FACS, and results are the averages from three independent experiments; the error bars are standard errors. (b) HEK293 cells were exposed to compound 1 (200 μM) or 10 μM actinomycin D (Act. D), a control for DNA fragmentation as a result of apoptosis, or left untreated. BrdU incorporation was analyzed as described for panel a. (c) Antitrypanosomal activity of the indicated compounds was tested in MMR-proficient (*MSH2*^*+/+*^) BF T. brucei or derived MMR-impaired lines lacking one (*MSH2*^+/−^) or both (*MSH2*^−/−^) alleles or the reexpressor line *MSH2*^−/−/+^. MNNG, *N*-methyl-*N*′-nitro-*N*-nitrosoguanidine; MMS, methyl methanesulfonate. *, *P* < 0.02; **, *P* < 0.005, as determined by a paired Student *t* test.

The cytostatic activity of anticancer diazomethane-releasing drugs, such as temozolomide and dacarbazine, is based on *O*^6^-guanine methylation and a consequent futile cycling of the DNA mismatch repair system (MMR), which ultimately results in DNA strand breaks and cell death by apoptosis (37). The MMR does not repair the *O*^*6*^-methylated guanine residue itself, but it is rather trying to correct the nucleotide that is base paired to it on the newly synthesized strand, and it thereby drives a futile cycle of repetitive nonproductive repair. To assess whether MTP treatment leads to DNA strand breaks in T. brucei, we performed a terminal deoxynucleotidyltransferase-mediated dUTP-biotin nick end labeling (TUNEL) assay, monitoring 5-bromo-2-deoxyuridine 5-triphosphate (BrdUTP) incorporation visualized by a fluorescein isothiocyanate (FITC)-labeled anti-BrdU antibody. Treatment of BF T. brucei with just 3 μM compound 1 for 24 h resulted in a 2-fold increase in BrdU incorporation, indicating that compound 1 induces DNA strand breaks (Fig. 4A). In contrast, exposure of human HEK cells to a much higher concentration of compound 1 (200 μM) for 24 h revealed no increase in BrdU incorporation relative to that of the controls, and thus no DNA strand breaks were induced (Fig. 4B).

Methyltriazenyl prodrugs release methyldiazonium species capable of methylating nucleobases by an S_N_1-type nucleophilic substitution mechanism, transferring the carbonium ion CH_3_^+^ to electron-rich centers, such as the exocyclic oxygen or nitrogen atoms of DNA bases (37). While S_N_1-type alkylators are MMR dependent, S_N_2-type alkylators (such as MMS and phleomycin) are not, as S_N_2-type alkylators predominantly target purine ring nitrogen atoms and generate double-strand breaks through base excision repair-mediated processing (38). Reduced expression or impairment of the MMR has been shown to confer resistance to DNA-alkylating agents of the S_N_1 type but not to those of the S_N_2 type (39, 40). The MMR machinery is well conserved throughout evolution, and trypanosomatids also have a functional MMR system (20, 21). The nuclear protein MSH2 is a central component of the eukaryotic MMR, and its inactivation leads to a loss of MMR function (41). In T. brucei, MSH2 mutants lacking one (*MSH2*^+/−^) or two (*MSH2*^−/−^) alleles were shown to be increasingly tolerant to DNA damage induced by the S_N_1-type alkylator *N*-methyl-*N*′-nitro-*N*-nitrosoguanidine (MNNG) (20, 21). We tested the toxicity of selected MTPs against four T. brucei cell lines, *MSH2*^*+/+*^ (wild-type), *MSH2*^+/−^ (single knockout), *MSH2*^−/−^ (double knockout), and *MSH2*^−/−/+^ (reexpressing [20]) cells and compared their activities to those of various reference S_N_1 and S_N_2 type alkylators. As expected, only S_N_1-type alkylators, the MTPs 1 and 2, temozolomide and MNNG, showed reduced potency against cells with impaired or lost MSH2 activity (see Table S1 in the supplemental material). Like MNNG, MTP 1 became significantly less effective against cells that have no MSH2 activity (*P* < 0.02 and <0.01 for single- and double-knockout cells, respectively) (Fig. 4C). Reintroduction of the *MSH2* gene reversed the decreased sensitivity. This indicated that the antitrypanosomal activity of compound 1 is, at least in part, mediated through base modifications recognized by the MMR machinery that lead to persistent DNA breaks.

## DISCUSSION

New drugs for African trypanosomiasis are needed, as current drugs are old, often toxic, and have become increasingly ineffective due to resistance (4, 6–8). A few new compounds are now proceeding to clinical test stages (7, 42), but trypanosomiasis is a complex set of diseases involving various pathogenic species, hosts, and disease stages for which a single panacea is unlikely to be developed. Using a rational drug design approach, we took advantage of the multiple high-affinity nucleobase uptake transporters in the T. brucei parasite for selective delivery of toxic cargo into the parasite. We synthesized a series of methyltriazenyl purine (MTP) prodrugs and evaluated their antitrypanosomal activities. We identified MTP derivative 1 with high antitrypanosomal potency and curative efficacy in an animal model of acute trypanosomiasis and investigated its mode of action.

**FIG 5:**
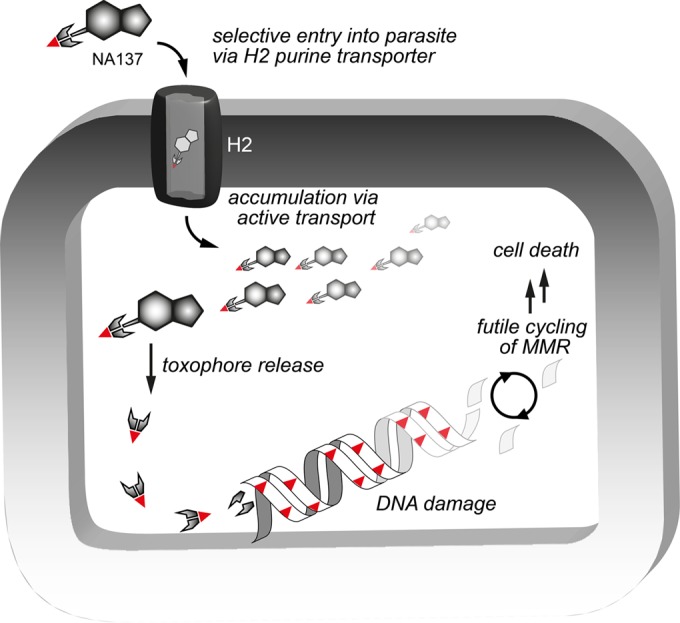
Proposed model of the mode of action of the methyltriazenyl purine-based drug 1. The purine moiety acts as a haptophore, through which the toxophore is delivered into the cell. Drug levels build up selectively in trypanosomes via highly efficient nucleobase transporters (proton symporters) in their cell membrane (H2 and H3). MTP compound 1 is hydrolyzed inside the parasite, with an approximate half-life of 6.8 h, releasing toxic methyldiazonium cations that cause DNA damage resulting in nucleotide mismatches during DNA replication above a lethal threshold level. Futile cycles of attempted mismatch repair lead to G_2_/M phase arrest and eventually cell death.

Based on our study, we suggest a working model for the parasite-selective toxicity displayed by compound 1 (Fig. 5). MTP 1 was designed to be taken up by active transport via the trypanosomal purine nucleobase transporters H2 and H3 (18). Indeed, compound 1 (and the derivative compound 2) revealed a significantly higher affinity for the trypanosomal H2 over the human FNT1 and displayed lower trypanocidal activity in medium containing high concentrations of hypoxanthine than the same concentration of inosine, consistent with competition at the level of uptake. Retention of potency against a drug-resistant line lacking the TbAT1/P2 aminopurine transporter gene indicated that this activity is not primarily involved in the uptake of MTPs, and cross-resistance with diamidine drugs is not likely to arise. This was in agreement with previous reports that TbAT1/P2 does not transport 6-oxopurines (43) and poorly tolerates even small substitutions on position 2 of aminopurines (44). Given the hydrophilic nature of compound 1**,** passive diffusion across any cell membrane, whether parasite or mammalian, is not likely to occur at a significant rate, which may, at least in part, account for the observed absence of mutagenicity, hepatotoxicity, and cytotoxicity against mammalian cells. We conclude that uptake through the T. brucei nucleobase transporters at least contributes to the selective cytotoxic activities of the MTPs reported here.

A high threshold concentration of S_N_1-type alkylating drugs seems to be required for inducing cell death in cancer cells, as implied by the minimum of 6,000 *O*^6^-methyl guanine lesions per cell (45, 46). The number of DNA lesions in trypanosomes leading to irrecoverable cell death remains to be determined, but active transport by purine transporters likely facilitates a high intracellular concentration of the S_N_1-type alkylator compound 1. In contrast, the high lethal threshold value is not likely to be attained in mammalian cells due to the requirement for MTP to be transported into the cytoplasm of the cell. Even if the MTPs were efficient substrates for the human facilitative nucleobase transporter (hFNT), this mechanism is nonconcentrative and merely facilitates the passive bidirectional passage across the plasma membrane (47). The observation that treatment with compound 1 induces double-strand breaks in T. brucei but not in human cells is consistent with its proposed mechanism of action and selectivity.

In fact, DNA damage may be a particular vulnerability of the trypanosome. Several drugs currently used in the clinic (nifurtimox-eflornithine combination therapy [NECT]) or in clinical trials (fexinidazole) against HAT (48) and leishmaniasis (49) have been shown to cause enhanced DNA lesions in trypanosomes. Eflornithine (difluoromethylornithine [DFMO]) causes a dramatic increase in decarboxylated *S*-adenosylmethionine (dSAM) and *S*-adenosylmethionine (SAM) in T. brucei *in vivo* (50), and these methylating metabolites may contribute to DNA alkylations (51). DFMO-induced depletion of trypanothione and polyamines may further potentiate the methylating activity of SAM by lowering levels of competing nucleophiles, while reduced levels of polyamines may make nucleic acids more susceptible to methylation (52). The nitro-heterocyclic prodrugs nifurtimox and fexinidazole (53) require bioreductive activation, which generates cytotoxic metabolites (54) that cause DNA (55), lipid, and protein damage (56).

DNA alkylation was proposed >2 decades ago as an antitrypanosomal strategy (57) but has since been neglected, as the alkylating species developed at that time, 1,2-bis(methylsulfonyl)-1-methylhydrazine, lacked parasite selectivity (51). We have revisited DNA alkylation as antiparasitic chemotherapy by exploiting the purine uptake system of the parasite for targeted delivery of a DNA-targeting toxin into the parasite and by optimizing the prodrug hydrolysis rate. We have shown that the MTP lead compound 1 acts on target, causing DNA strand breaks in T. brucei but not in human HEK cells. MTP compound 1 is well tolerated (up to 10 × 50 mg/kg i.p. daily without any overt adverse effects) and cures acute trypanosomiasis in mice while showing a high cure rate in acute T. brucei rhodesiense infection *in vivo*, superior to that of pentamidine (58). The observed absence of genotoxicity, hepatotoxicity, and cytotoxicity against mammalian cells revitalizes the idea of pursuing DNA alkylators as a safe chemotherapeutic option for the treatment of human or veterinary trypanosomiasis.

## Supplementary Material

Supplemental material
