# Supplementary material for: Targeting the Parasite's DNA with Methyltriazenyl Purine Analogs Is a Safe, Selective, and Efficacious Antitrypanosomal Strategy
Source: Antimicrob Agents Chemother. 2015 Oct 13;59(11):6708–16. doi: 10.1128/AAC.00596-15 (PMC4604408; doi:10.1128/AAC.00596-15)
Supplement: Supplemental material [file supp_59_11_6708__index.html]

Supplemental material 

# Targeting the Parasite's DNA with Methyltriazenyl Purine Analogs Is a Safe, Selective, and Efficacious Antitrypanosomal Strategy

## Supplemental material

- Supplemental file 1 -

  Supplemental text, Figures S1 to S8, and Table S1.

  PDF, 751K
