# Supplementary material for: Targeting the Parasite's DNA with Methyltriazenyl Purine Analogs Is a Safe, Selective, and Efficacious Antitrypanosomal Strategy
Source: Antimicrob Agents Chemother. 2015 Oct 13;59(11):6708–16. doi: 10.1128/AAC.00596-15 (PMC4604408; doi:10.1128/AAC.00596-15)
Supplement: Supplemental material [file AAC.00596-15_zac011154513so1.pdf]

Supplementary Data

to the article entitled:

Targeting the parasite's DNA with methyltriazenyl purine analogs is a safe, selective and efficacious antitrypanosomal strategy.

Boris Rodenko, Martin J. Wanner, Abdulsalam A. M. Alkhaldi, Godwin U. Ebiloma, Rebecca L. Barnes, Marcel Kaiser, Reto Brun, Richard McCulloch, Gerrit-Jan Koomen and Harry P. de Koning.

Contents	Page
**Supplementary Data Text describing the synthesis and characterization of triazenyl purines**	S2
**Figure S1**	S9
**Figure S2**	S10
**Figure S3**	S11
**Figure S4**	S12
**Figure S5**	S13
**Figure S6**	S14
**Figure S7**	S15
**Figure S8**	S16
**Table S1**	S17
**Supplementary Data References**	S18

Supplementary Data Text

Chemistry.

General remarks. All ^{1}H NMR and ^{13}C NMR spectra (APT) were recorded with a Bruker Avance 400 spectrometer (^{1}H 400 MHz, ^{13}C 100 MHz) at room temperature in the solvent indicated. Analytical thin layer chromatography was performed using a Merck TLC plastic roll 500 x 20 cm silica gel 60 F_{254}. Flash chromatography was performed on Biosolve 60 Å (0.032-0.063 mm) silica gel. Melting points were measured with a Leitz-Wetzlar melting point microscope apparatus and are uncorrected. ESI mass spectra were recorded in ES+ mode on an LCT™ Orthogonal Acceleration Time of Flight Mass Spectrometer (Waters).

Synthesis of methyltriazenyl purines

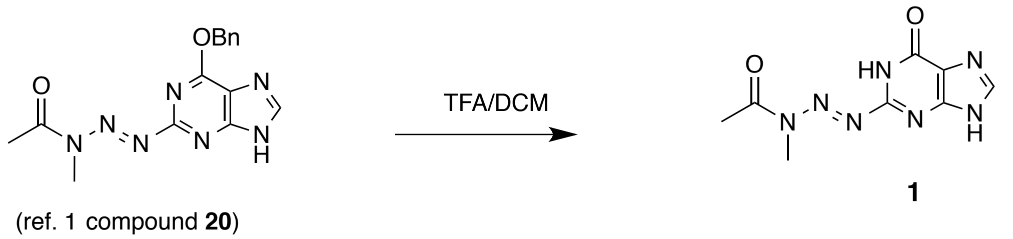

2-(3-Acetyl-3-methyltriazen-1-yl)-6-hydroxypurine (1). A solution of 2-(3-acetyl-3-methyltriazen-1-yl)-6-benzyloxypurine (1) (0.35 g; 1.1 mmol) in 20 mL DCM-TFA (3:1) was stirred at room temperature for 17 h. The reaction mixture was coevaporated with toluene (2x10 mL) to dryness and the residue was triturated with 6 mL CH_3CN-Et_2O (5:1). The product was obtained by filtration and dried *in vacuo*, yielding a light-yellow solid (0.20 g; 0.85 mmol; 77%), mp > 180 °C (dec); ^{1}H NMR (DMSO-d6) δ 12.44 (s, 1H); 8.21 (s, 1H); 3.36 (s, 3H); 2.59 (s, 3H); ^{13}C NMR (DMSO-d6) δ 174.07, 156.26, 153.74, 142.02, 118.97, 29.23, 22.72; [found $[M+H]^{+1}$, 236.04; $C_8H_9N_7O_2$ requires, 236.02].

^{1}H-NMR spectrum of **1** in DMSO-d6.

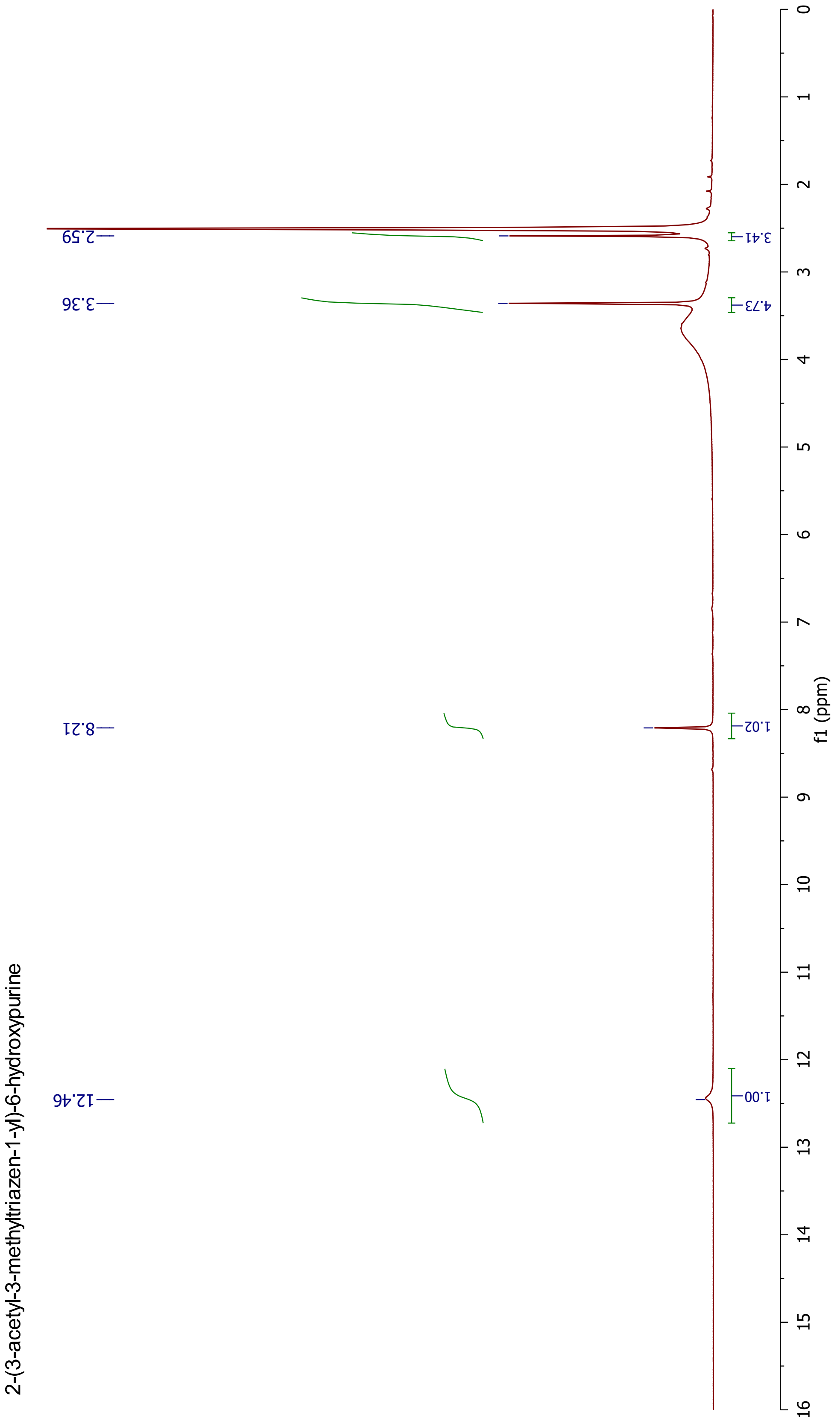

^{13}C-APT NMR spectrum of **1** in DMSO-d6.

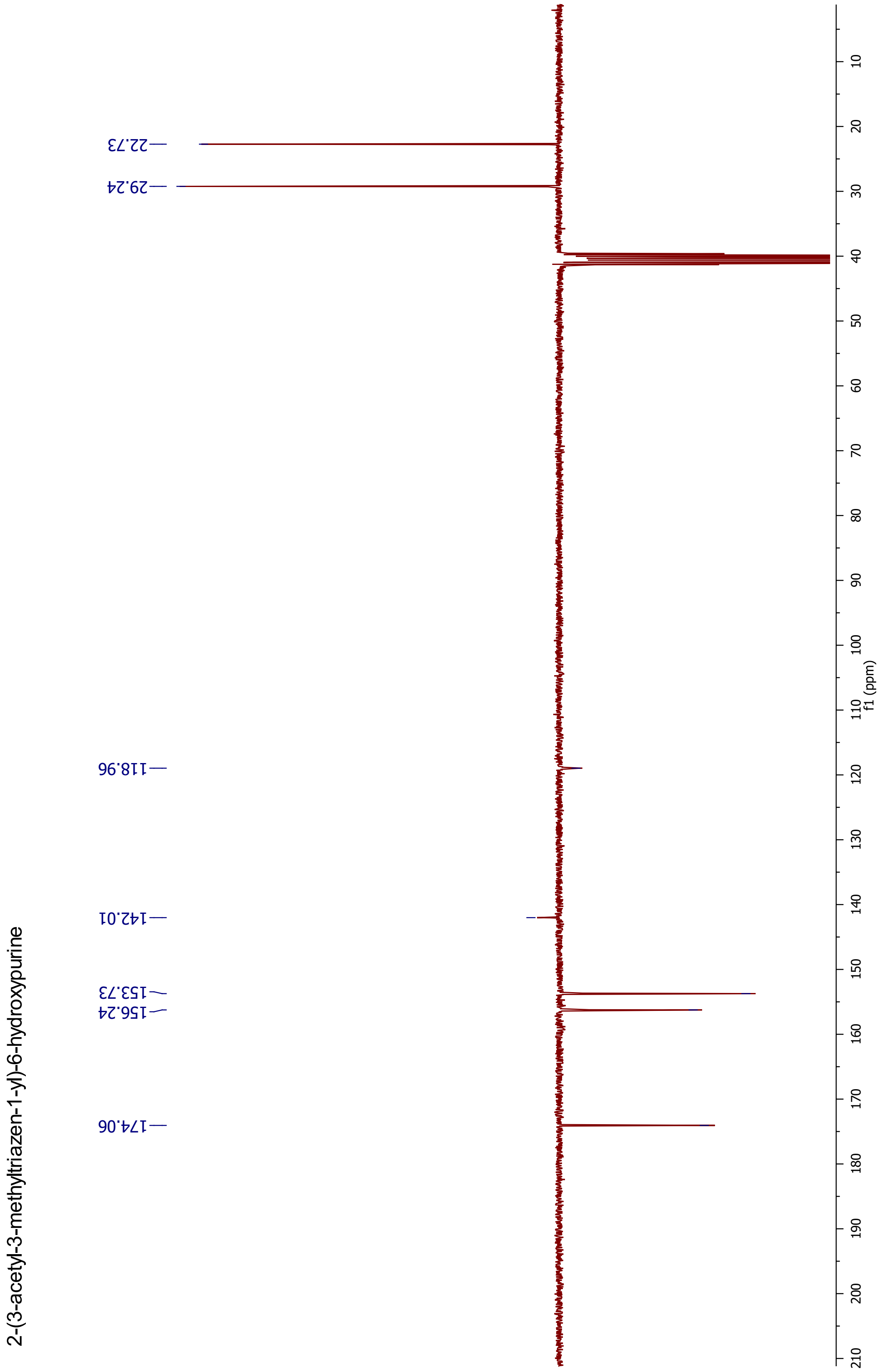

ESI MS spectrum of **1** measured at cone voltage 30 V, desolvation temperature 250 °C, source temperature 120 °C.

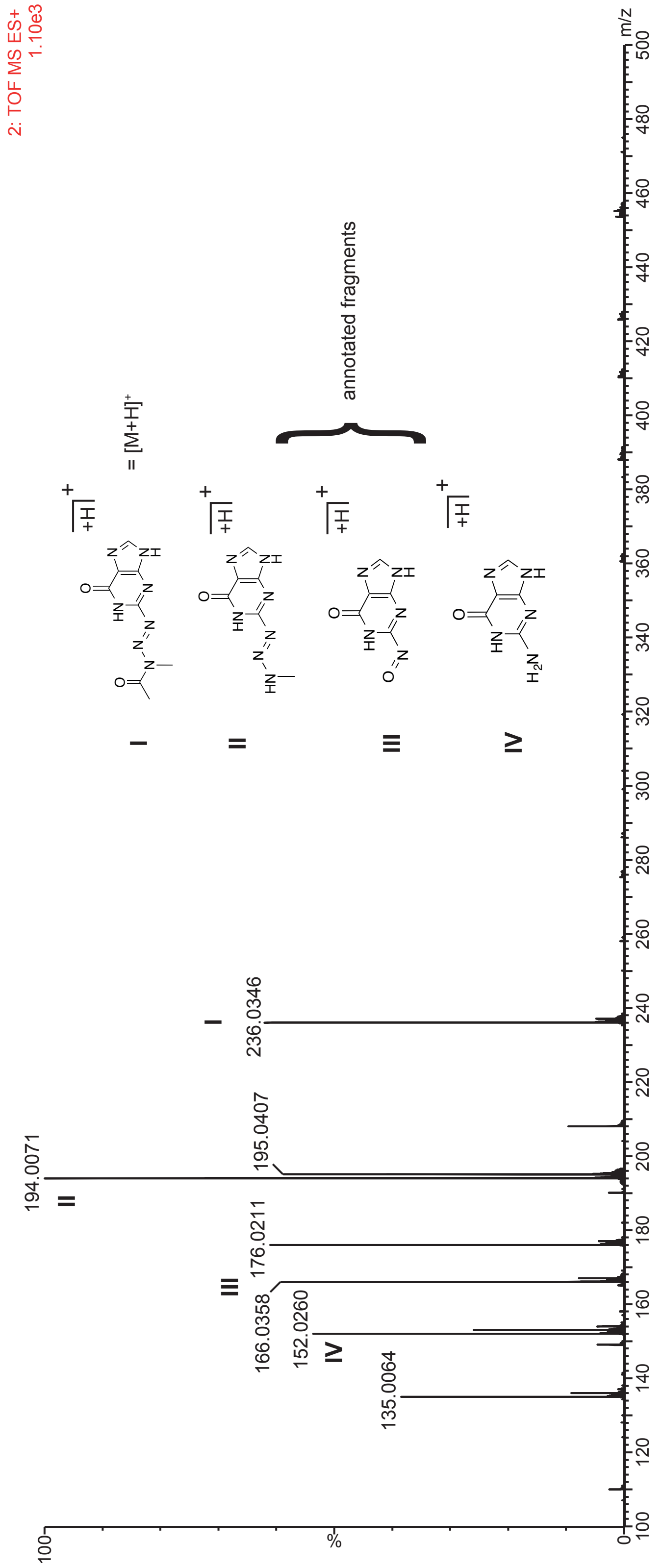

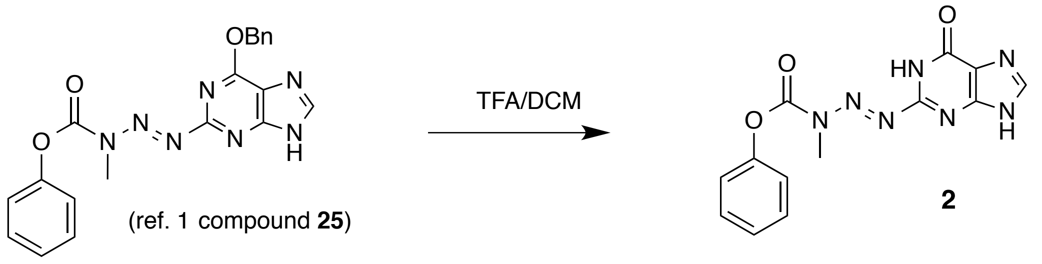

2-(3-Phenoxycarbonyl-3-methyltriazen-1-yl)-6-hydroxypurine (2). A solution of 2-(3-phenoxycarbonyl-3-methyltriazen-1-yl)-6-benzyloxypurine (1) (0.202 g; 0.50 mmol) in 4 mL DCM-TFA (3:1) was stirred at room temperature for 17 h. The reaction mixture was coevaporated with toluene (2x10 mL) to dryness and the residue was recrystallized from CH_3CN. The product was obtained by filtration and dried *in vacuo*, yielding a light-yellow solid (0.151 g; 0.48 mmol; 96%), mp > 280 °C (dec); ^{1}H NMR (DMSO-d6) δ 13.0 (bs, 1H); 12.68 (s, 1H); 8.19 (s, 1H); 7.50 – 7.54 (m, 2H); 7.35 – 7.38 (m, 3H); 3.55 (s, 3H).

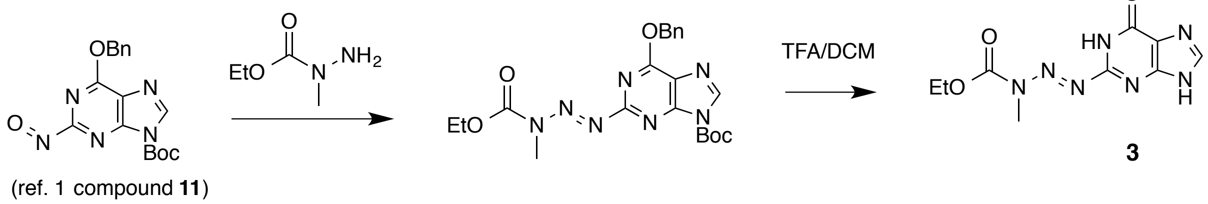

2-(3-Ethoxycarbonyl-3-methyltriazen-1-yl)-6-hydroxypurine (3). A solution of 2-(3-ethoxycarbonyl-3-methyltriazen-1-yl)-6-benzyloxy-9-Boc-purine (1) (0.062 g; 0.14 mmol) in 1.5 mL DCM-TFA (3:1) was stirred at room temperature for 17 h. The reaction mixture was coevaporated with toluene (2x5 mL) to dryness and the residue was recrystallized two times from CH_3CN. The product was obtained by filtration and dried *in vacuo*, yielding a light-yellow solid (0.025 g; 0.11 mmol; 76%), mp 168 – 178 °C (dec); ^{1}H NMR (DMSO-d6) δ 13.5 (bs, 1H); 12.6 (s, 1H); 8.17 (s, 1H); 4.40 (q, 1H, J = 7.1 Hz); 3.41 (s, 3H); 1.32 (t, 3H, J = 7.1 Hz).

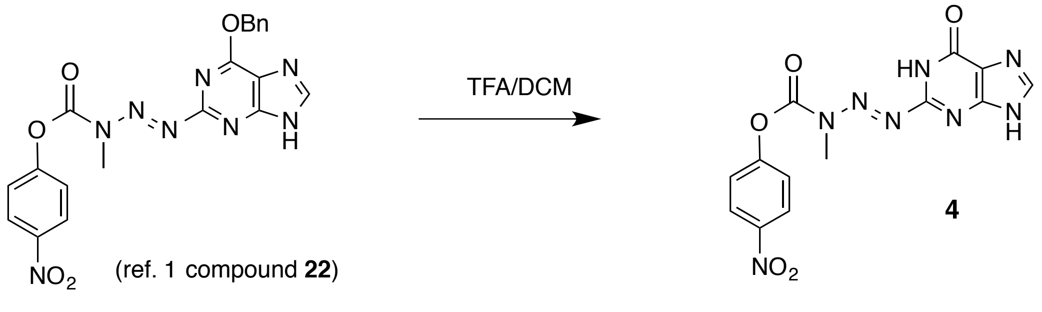

2-(3-*p*-Nitrophenoxycarbonyl-3-methyltriazen-1-yl)-6-hydroxypurine (4). A solution of 2-(3-*p*-nitrophenoxycarbonyl-3-methyltriazen-1-yl)-6-benzyloxypurine (1) (0.070 g; 0.156 mmol) in 4 mL DCM-TFA (3:1) was stirred at room temperature for 17 h. The reaction mixture was coevaporated with toluene (2x10 mL) to dryness and the residue was first triturated with diethyl ether (10 ml) and finally recrystallized from CH_3CN. The product was dried *in vacuo*, yielding a yellow solid (0.040 g; 0.111 mmol; 71%), mp > 280 °C (dec); ^{1}H NMR (DMSO-d6) δ 12.5 (br, 1H); 10.8 (s, 1H); 8.38 – 8.43 (m, 2H); 8.26 (s, 1H); 7.68 – 7.72 (m, 2H); 3.56 (s, 3H).

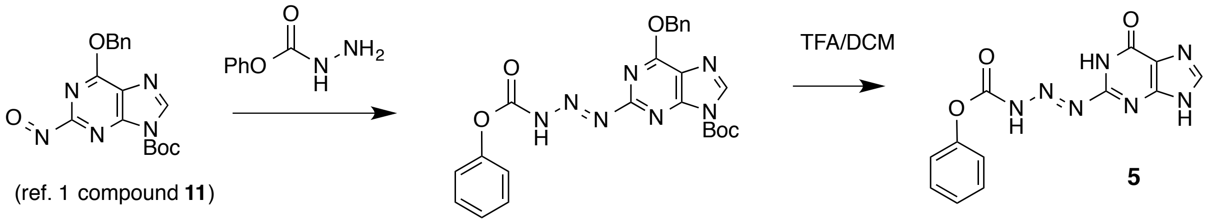

2-(3-Phenoxycarbonyl-triazen-1-yl)-6-benzyloxy-9-Boc-purine. A solution of 2-nitroso-6-benzyloxy-9-Boc-purine (1) (0.071 g; 0.2 mmol) and phenyl carbazate (0.037 g, 0.24 mmol) in 2 mL DCM-acetic acid (3:1) was stirred at room temperature for 2 h. Extractive workup (aqueous $NaHCO_3$, DCM) and crystallization from ethyl acetate gave the product (0.060 g, 0.122 mmol, 61%), mp 146 – 149 °C (dec); ^{1}H NMR ($CDCl_3$) *δ* 12.30 (s, 1H) 7.97 (s, 1H); 7.55 – 7.25 (m, 10H); 5.53 (s, 2H); 1.69 (s, 9H).

2-(3-Phenoxycarbonyl-triazen-1-yl)-6-hydroxypurine (5). A solution of 2-(3-phenoxycarbonyl-triazen-1-yl)-6-benzyloxy-9-Boc-purine (0.055 g; 0.23 mmol) in 1.5 mL DCM-TFA (3:1) was stirred at room temperature for 17 h. The reaction mixture was coevaporated with toluene (2x5 mL) to dryness and the residue was triturated with CH_3CN. The product was obtained by filtration and dried *in vacuo*, yielding the product as a solid (0.151 g; 0.48 mmol; 96%), mp > 280 °C (dec); ^{1}H NMR (DMSO-d6) *δ* 13.0 (bs, 1H); 12.68 (s, 1H); 8.19 (s, 1H); 7.50 – 7.54 (m, 2H); 7.35 – 7.38 (m, 3H).

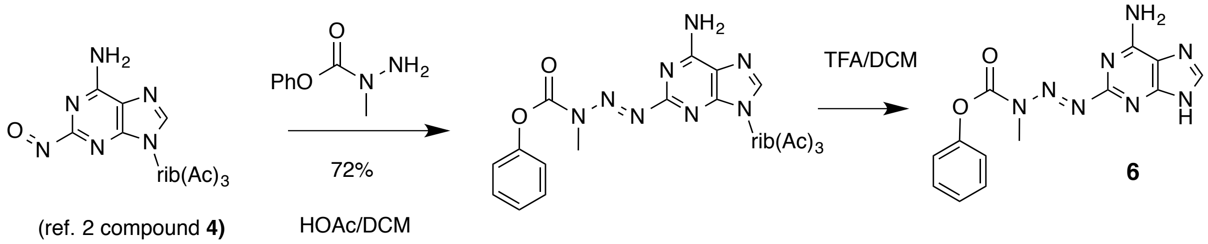

2′,3′,5′-Tri-*O*-acetyl-2-(3-phenoxycarbonyl-3-methyltriazen-1-yl)-adenosine. A solution of 2′,3′,5′-tri-*O*-acetyl-2-nitrosoadenosine (2) (0.211 g; 0.5 mmol) and phenylcarbamoyl-1-methylhydrazine (1) (0.100 g, 0.6 mmol) in 3.0 mL DCM-acetic acid (5:1) was stirred at room temperature for 2 h. Extractive workup (aqueous $NaHCO_3$, DCM) and chromatography (silica, ethyl acetate/methanol 97/3) gave the product (0.206 g, 0.362 mmol, 72 %) as a glass. ^{1}H NMR ($CDCl_3$) *δ* 7.97 (s, 1H); 7.41 (m, 2H); 7.27 (m, 3H); 6.26 (bs, 2H); 6.24 (d, 1H, *J* = 5.1 Hz); 5.80 (m, 1H); 5.68 (m, 1H), 4.40 - 4.41 (m, 3H); 3.67 (s, 3H); 2.07, 2.07, and 2.04 (all 3H, s).

2-(3-Phenoxycarbonyl-3-methyltriazen-1-yl)-6-aminopurine (6). A solution of 2′,3′,5′-tri-O-acetyl-2-(3-phenoxycarbonyl-3-methyltriazen-1-yl)-adenosine (0.145 g, 0.255 mmol) in TFA (2 ml) was stirred at 48 °C for 17 h. The reaction mixture was coevaporated with toluene (2x10 mL) to dryness and the residue was first triturated with diethyl ether (10 ml) and finally triturated with hot CH_3CN to give the product (0.068 g, 0.22 mol, 85%) as a light-yellow solid, mp > 280 °C (dec); ^{1}H NMR (DMSO-d6) *δ* 8.29 (s, 1H), 7.60 (bs, 2H); 7.48 – 7.50 (m, 2H); 7.33 – 7.37 (m, 3H); 3.51 (s, 3H).

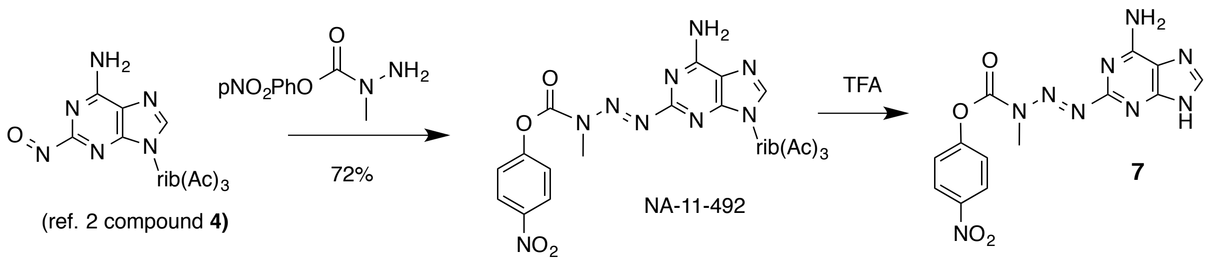

2′,3′,5′-Tri-*O*-acetyl-2-(3-*p*-nitrophenoxycarbonyl-3-methyltriazen-1-yl)-adenosine. A solution of 2′,3′,5′-tri-*O*-acetyl-2-nitrosoadenosine (2) (0.105 g; 0.25 mmol) and 1-*p*-nitrophenylcarbamoyl-1-methylhydrazine (1) (0.063 g, 0.3 mmol) in 2.5 mL DCM-acetic acid (5:1) was stirred at room temperature for 2 h. Extractive workup (aqueous $NaHCO_3$, DCM) and chromatography (silica, ethyl acetate/methanol 98/2) gave the product (0.102 g, 0.166 mmol, 66%) as a glass. ^1H NMR ($CDCl_3$) δ 8.25 (m, 2H); 7.99 (s, 1H); 7.45 (m, 2H); 6.70 (bs, 2H); 6.22 (d, 1H, J = 5.1 Hz); 5.87 (m, 1H); 5.66 (m, 1H); 4.39 - 4.41 (m, 3H); 3.64 (s, 3H); 2.06, 2.05, and 2.03 (all 3H, s).

2-(3-*p*-Nitrophenoxycarbonyl-3-methyltriazen-1-yl)-6-aminopurine (7). A solution of 2′,3′,5′-tri-O-acetyl-2-(3-*p*-nitrophenoxycarbonyl-3-methyltriazen-1-yl)-adenosine (0.102 g, 0.166 mmol) in TFA (2 ml) was stirred at 48 °C for 17 h. The reaction mixture was coevaporated with toluene (2x10 mL) to dryness and the residue was first triturated with diethyl ether (10 ml) and finally triturated with CH_3CN to give the product (0.038 g, 0.106 mmol, 64%) as a yellow solid, mp > 280 °C (dec); ^1H NMR (DMSO-d6) δ 8.38 (m, 2H), 8.30 (s, 1H); 7.66 (m, 2H); 7.60, bs, 2H); 3.52 (s, 3H).

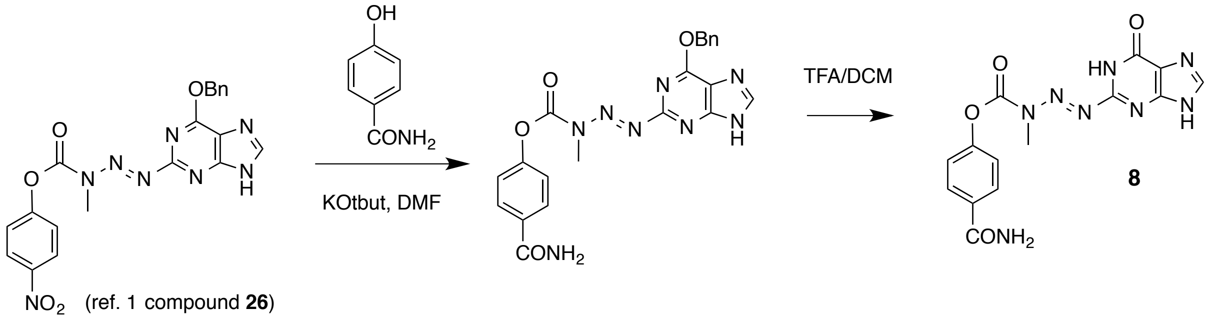

2-(3-*p*-Carbamoylphenoxycarbonyl-3-methyltriazen-1-yl)-6-benzyloxy-purine. Potassium-*t*-butoxide (0.034 mg, 0.3 mmol) was added to a solution of *p*-hydroxybenzamide (0.041 g, 0.3 mmol) in anhydrous DMF (1ml). To this anion was added 2-(3-*p*-nitrophenoxycarbonyl-3-methyltriazen-1-yl)-6-benzyloxypurine (1) (0.045 g, 0.1 mmol) and after 2 h stirring the mixture was acidified with acetic acid (0.050 g). Addition of water (1 ml) and recrystallization of the resulting solid from DMF/water gave a yellow solid (0.029 g, 0.065 mmol, 65%), mp 210 – 212 °C; ^1H NMR ($CDCl_3$) δ 13.6 (bs, 1H); 8.44 (s, 1H); 8.05 (s, 1H); 8.00 (d, 2H, J = 8.5 Hz); 7.3 – 7.6 (m, 7H); 5.67 (s, 2H,); 3.58 (s, 3H).

2-(3-*p*-Carbamoylphenoxycarbonyl-3-methyltriazen-1-yl)-6-hydroxy-purine (8). A solution of 2-(3-*p*-carbamoylphenoxycarbonyl-3-methyltriazen-1-yl)-6-benzyloxypurine (0.029 g; 0.065 mmol) in 1.5 mL DCM-TFA (3:1) was stirred at room temperature for 17 h. The reaction mixture was coevaporated with toluene (2x5 mL) to dryness and the residue was triturated with CH_3CN. The product was dried *in vacuo*, yielding a yellow solid (0.016 g; 0.055 mmol; 84%), mp > 280 °C (dec); ^1H NMR (DMSO-d6) δ 12.3 (bs, 1H); 10.2 (s, 1H); 8.10 (s, 1H); 7.80 (d, 1H, J = 8.5 Hz); 6.80 (d, 1H, J = 8.5 Hz); 3.50 (s, 3H).

Figure S1

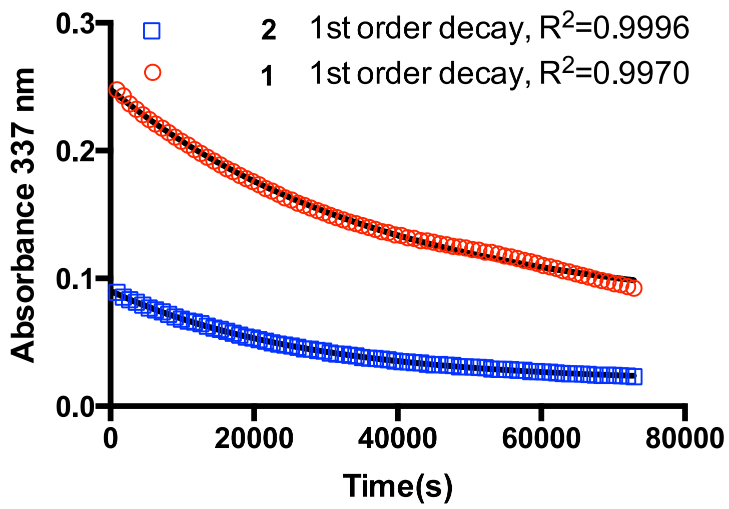

Figure S1. Triazene hydrolysis is responsible for the release of methyldiazonium cations. The rate of spontaneous hydrolysis of the indicated methyltriazenyl purine at 50 µM in PBS at 37 °C was determined by UV-spectroscopy. Exponential decay of the triazenyl absorption maximum at 337 nm indicated a first order reaction with a high goodness of fit, R^2>0.99. A representative hydrolysis experiment is shown. The half-life of **2** = 5.6 ± 0.3 h; the half life of **1** = 6.8 ± 0.4 h (n=2).

Figure S2

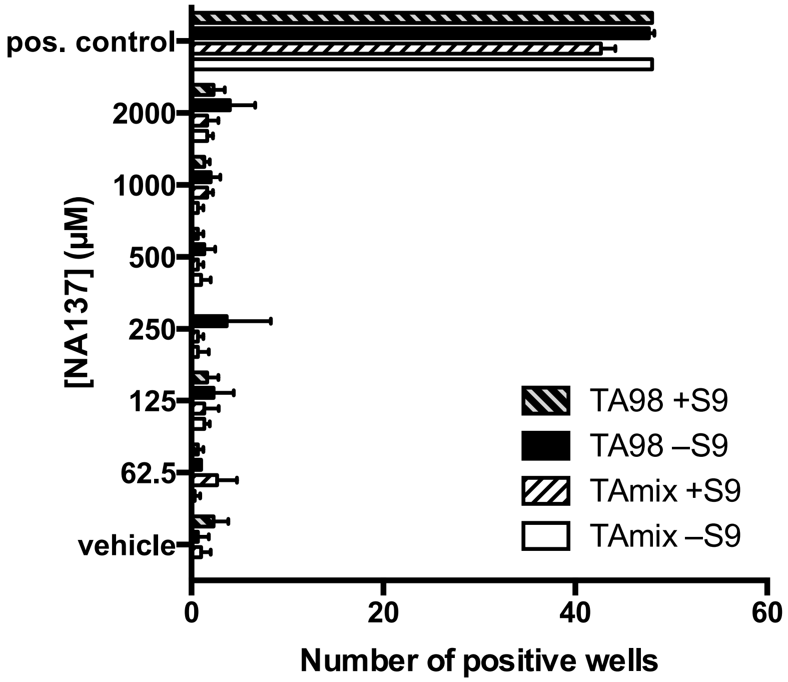

Figure S2. Methyltriazenyl purine 1 shows no mutagenic activity in the Ames II test. *In vitro* mutagenic activity of **1** was tested by NOTOX BV ('s Hertogenbosch, The Netherlands) using the Ames II Mutagenicity assay kit in microtiter plate format in compliance with the NOTOX Standard Operating Procedure GEN/C/526 and following the manufacturer's protocol (Xenometrix AG, Allschwil, Switzerland) (3). Specific point mutations in the histidine operon in *Salmonella typhimurium* renders these bacteria incapable of producing histidine. These His^{-} bacteria are unable to grow on medium lacking histidine. The Ames II assay scores for bacterial growth after incubation with test compound in wells containing His– medium as an indication of reversal of these point mutations. His– *S. typhimurium* strains TA98 were used for the detection of frameshift mutations and TAMix, consisting of 6 histidine mutant tester strains, TA7001-TA7006, for the detection of base pair mutations. For testing mutagenicity due to mammalian metabolic activation, the test was performed in the absence or presence of an exogenous metabolic activation system of the Aroclor 1254 induced rat-liver homogenate fraction S9. MTP **1** was tested in 6 doubling dilutions ranging from 2 mM to 62.5 µM and scored negative (not mutagenic) both before (–S9) and after (+S9) metabolic activation. Vehicle = dmso; positive controls without S9: TAmix, 4-nitroquinoline-*N*-oxide (0.5 µg/mL); TA98, 2-nitrofluorene (2 µg/mL); TAmix or TA98 with S9: 2-aminoanthracene (5 µg/mL).

Figure S3

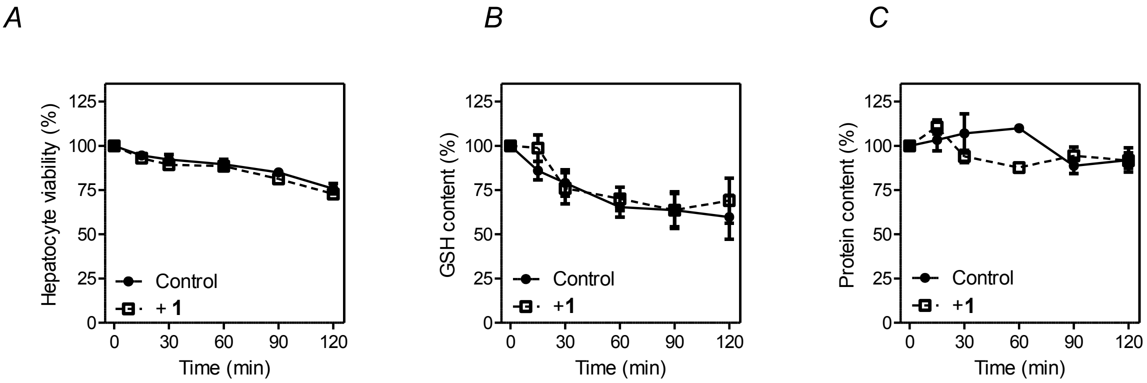

Figure S3. MTP 1 does not show toxic effects on freshly isolated rat hepatocytes. (*A*) Hepatocytes from male Sprague-Dawley rats were isolated as described (4, 5) and hepatotoxicity assays were performed as described (6). Cells were treated with 100 µM of **1** or left untreated. Cell viability was determined by a Trypan blue exclusion test. Values are normalized to t=0 min (%). (*B*) The reduced glutathione (GSH) content of hepatocytes was determined by an *O*-phthaldehyde fluorimetric test. Values are normalized to t=0 min (%). Abnormal changes in GSH levels, indicative of cellular stress, were not observed upon treatment with **1**. (C) The total protein content of hepatocytes was determined by a Lowry assay. Values are normalized to t=0 min (%). No protein loss through reduced cell integrity is observed upon treatment with **1**.

Figure S4

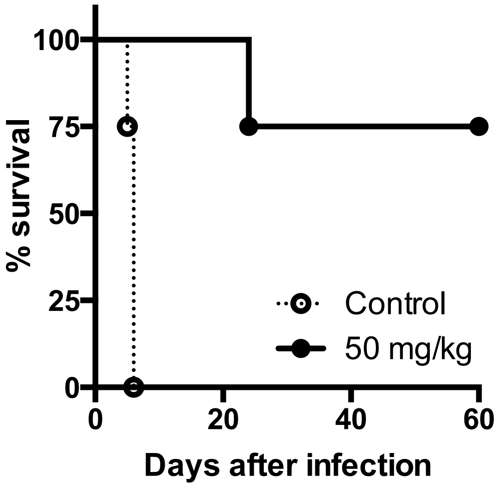

Figure S4. Methyltriazenyl purine 1 has a high cure rate in acute *T. b. rhodesiense infection in vivo*. Kaplan–Meier survival plot for female NMRI mice (n = 4 per group) after infection with *T. b. rhodesiense* (STIB900) (inoculum 3×10^3 parasites). Intraperitoneal injection with **1** dissolved in DMSO started 3 days after infection at a single dose of 50 mg/kg per day for 4 days.

Figure S5

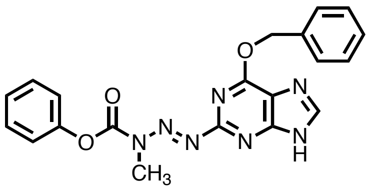

10

Trypanosoma brucei rhodesiense:

EC_{50} = 8.9 μM

Plasmodium falciparum: EC_{50} >10 μM

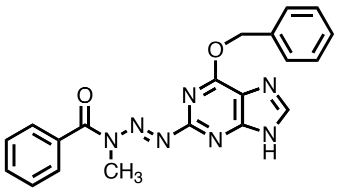

11

Trypanosoma brucei rhodesiense:

EC_{50} = 9.6 μM

Plasmodium falciparum: EC_{50} = 12.8 μM

Figure S5. Antitumor *O*^{6}-benzyl-2-methyltriazenylpurines with moderate antiprotozoal activity. Antiprotozoal evaluation on *T. b. rhodesiense* STIB 900 and *P. falciparum* K1 strain was determined exactly as described previously (7).

Figure S6

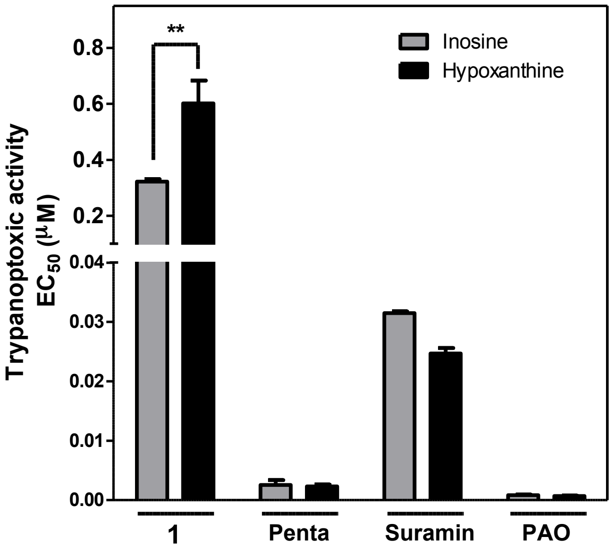

Figure S6. Antitrypanosomal activity of methyltriazenyl purine 1 is reduced in the presence of hypoxanthine, implicating H2/H3 transporter mediated uptake of 1. Effect of **1** and a set of control trypanocides on wild type *T. b. brucei*. Cells were grown in CMM with either inosine or hypoxanthine as the sole purine source, as indicated. Data are the average of 5 independent determinations and SE. **, P<0.02, unpaired Student's t-test.

Figure S7

A

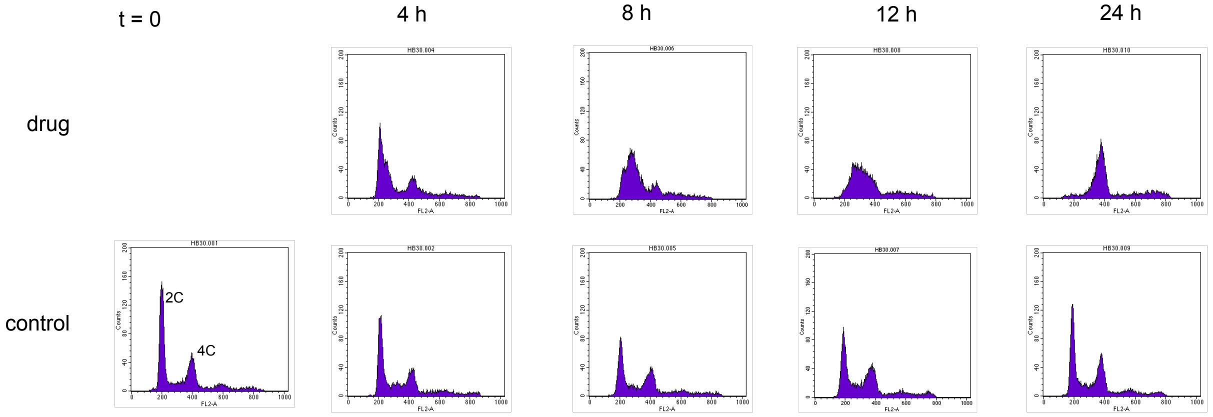

B

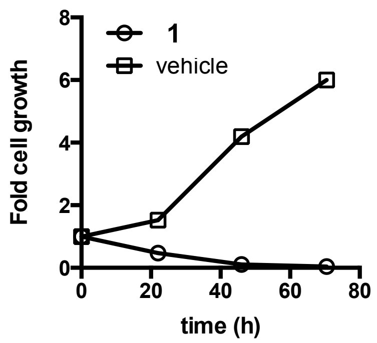

Figure S7. Methyltriazenyl purines cause cell cycle arrest in the G2/M phase. A) Flow cytometry analysis of bloodstream form *T.b. brucei* s427 cultures incubated in the presence or absence (controls) of 15 μM of **1**. Cells were fixed with 70% methanol and treated with RNase prior to staining with PI and analysis by flow cytometry. Each panel represents the counting of 10,000 cells. B) Long term growth curve of bloodstream form *T.b. brucei* s427 cultures (seeded at 5 × 10^5 cells/mL) exposed to 15 μM of **1** (closed squares) or vehicle only (open circles) monitored for 72 hours. Cell growth is arrested and the cells eventually die.

Figure S8

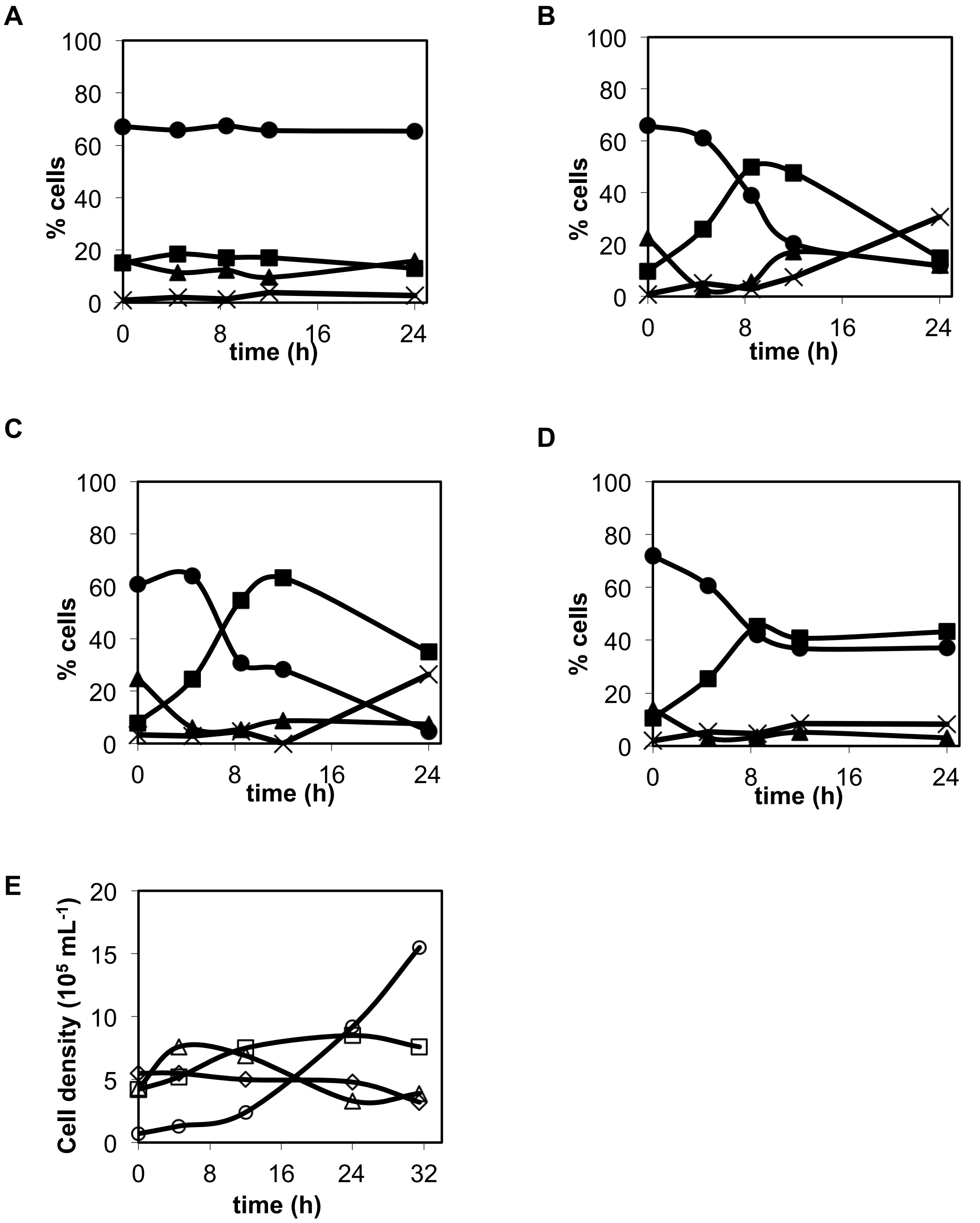

Figure S8. Methyltriazenyl purine 1 causes cell cycle and cytokinesis defects. Karyotype distribution of bloodstream form T. brucei (s427) exposed to vehicle (A) or 5 µM (B), 15 µM (C) or 50 µM (D) of **1** monitored over time; filled circles, 1N1K; filled squares, 1N2K; filled triangles, 2N2K; crosses, other karyotypes. Drug treated cells were arrested in cell growth and corresponding cell densities are shown in panel E, open circles, vehicle; open squares, 5 µM; open triangles, 15 µM; open diamonds, 50 µM. Cells were counted at the indicated times using a haemocytometer.

Supporting Information Table S1

Table S1. In vitro activity of methyltriazenyl purines, alkylating agents and trypanocides against control *Trypanosoma brucei* (*msh2*$^{+/+}$), and derived lines lacking one (*msh2*$^{+/-}$) or both (*msh2*$^{-/-}$) MSH2 alleles.[a]

		**Tbb *msh2*$^{+/+}$ (EC$_{50}$; μM)**		**Tbb *msh2*$^{+/-}$ (EC$_{50}$; μM)**		**Tbb *msh2*$^{-/-}$ (EC$_{50}$; μM)**	
**Drug**	**Type of drug**	**AVG**	**SE**	**AVG**	**SE**	**AVG**	**SE**
**1**	S_N1 alkylator	0.87	0.07	1.5	0.09	3.9	0.3
**2**	S_N1 alkylator	2.3	0.7	4.3	1.4	6.8	1.9
**5**	non-alkylator	192	29	166	34	168	34
**MNNG**	S_N1 alkylator	3.3	0.8	8.6	2.5	22.4	7.4
**TMZ**	S_N1 alkylator	95	7	204	32	173	44
**Cisplatin**	DNA cross-linker	13.4	6.2	17.7	10.1	13.6	8.5
**MMS**	S_N2 alkylator	48.9	7.2	57.2	3.9	46.7	2.3
**Phleomycin**	S_N2 alkylator	0.20	0.02	0.11	0.07	0.068	0.03
**diminazene**	trypanocide	0.50	0.12	0.64	0.18	0.68	0.27
**PAO**	trypanocide	0.00151	0.00022	0.0016	0.00035	0.00093	0.00027
**Pentamidine**	trypanocide	0.011	0.001	0.011	0.003	0.0040	0.0010

[a)] Drug efficacies, expressed as EC$_{50}$ values, were determined using an Alamar Blue assay, exactly as described (8, 9). AVG, average of at least 3 independent experiments; SE, standard error. TMZ is temozolomide, MNNG is 1-methyl-3-nitro-1-nitrosoguanidine, MMS is methylmethanesulfonate, PAO is phenylarsine oxide.

Supporting Information References

1. Wanner MJ, Koch M, Koomen G-J (2004) Synthesis and Antitumor Activity of Methyltriazene Prodrugs Simultaneously Releasing DNA-Methylating Agents and the Antiresistance Drug O6-Benzylguanine. *J Med Chem* 47:6875–6883.

2. Wanner MJ, Koomen G-J (2001) Synthesis and properties of 2-nitrosoadenosine. *J Chem Soc, Perkin Trans 1*:1908–1915.

3. Flückiger-Isler S et al. (2004) Assessment of the performance of the Ames II™ assay: a collaborative study with 19 coded compounds. *Mutat Res, Genet Toxicol Environ Mutagen* 558:181–197.

4. Moldéus P, Högberg J, Orrenius S (1978) Isolation and use of liver cells. *Methods Enzymol* 52:60–71.

5. Omar K, Grant MH, Henderson C, Watson DG (2013) The abundant dietary constituent ferulic acid forms a wide range of metabolites including a glutathione adduct when incubated with rat hepatocytes. *Xenobiotica*:1–6.

6. Jairaj M, Watson DG, Grant MH, Skellern GG (2003) The toxicity of opiates and their metabolites in HepG2 cells. *Chem-Biol Interact* 146:121–129.

7. Rodenko B et al. (2007) 2,N6-disubstituted adenosine analogs with antitrypanosomal and antimalarial activities. *Antimicrob Agents Chemother* 51:3796–3802.

8. Räz B, Iten M, Grether-Buhler Y, Kaminsky R, Brun R (1997) The Alamar Blue assay to determine drug sensitivity of African trypanosomes (*T.b. rhodesiense* and *T.b. gambiense*) in vitro. *Acta Trop* 68:139–147.

9. Wallace LJM, Candlish D, de Koning HP (2002) Different substrate recognition motifs of human and trypanosome nucleobase transporters. Selective uptake of purine antimetabolites. *J Biol Chem* 277:26149–26156.
